# Innovations in chemotherapy and radiation therapy: Implications and opportunities for the Asia-Pacific Rim

**DOI:** 10.2349/biij.4.3.e40

**Published:** 2008-07-01

**Authors:** DE Heron, JE Shogan, JW Mucenski

**Affiliations:** 1 Department of Radiation Oncology, University of Pittsburgh Cancer Institute, Pittsburgh, Pennsylvania, United States; 2 Department of Medical Oncology, UPMC Cancer Centers, Pittsburgh, Pennsylvania, United States

**Keywords:** Cancer, radiation therapy, chemotherapy, supportive care

## Abstract

New cases of invasive cancer in the United States occur among nearly 1.5 million people annually. In 2007, more than 1,500 people died per day with this diagnosis. Cancer is responsible for nearly one in every four deaths reported in the country. Enormous amounts of money and research have been, and are being spent, in an attempt to improve these numbers. While prevention and early detection remain the key to long-term success, treatment in the neo-adjuvant, adjuvant and metastatic settings still centre around two main treatment modalities – radiation therapy and chemotherapy. This article will review the advances that have been made in both areas that are making these treatments more precise and convenient, as well as less toxic, for the patient. In the field of radiation therapy this involves the development of new therapy planning and delivery systems, such as intensity-modulated radiation therapy (IMRT), and positron emission and computed tomography, PET-CT. Chemotherapy has also evolved with the development of targeted chemotherapy for the treatment of specific malignancies as well as improved supportive care agents which allow for the administration of dose-dense chemotherapy when appropriate.

## INTRODUCTION

The number of new cancer patients diagnosed in the United States in 2007 was 1,444,920, excluding patients with carcinoma in situ (except of the bladder) and either squamous or basal cell carcinoma of the skin. The number of deaths attributed to a cancer diagnosis in 2007 was 559,650, or more than 1,500 people per day. Cancer is second only to heart disease as the leading cause of death in the country and is responsible for 1 in every 4 deaths [1].

While those statistics are quite grim, the 5-year survival rate for all cancers diagnosed was reported to be 66% in the last years of reporting – 1996 through 2002 – which is up substantially from the mid-1970’s when the number hovered around 50% [1]. Unfortunately, these improvements are not noted in every cancer, nor in every patient based upon age, race or sex. There are a number of reasons for this dramatic improvement including, but not limited to, the advances in detection and treatment of the disease as well as improved supportive care drugs and an improved understanding of the molecular changes which may contribute to the development of a malignancy. These numbers will only improve when the data since 2002 becomes available.

The most common malignancies for women in the U.S. as of 2007 are breast cancer (178,480), lung cancer (98,620) and colorectal cancer (74,630). The death rates associated with these malignancies are 70,880 patients with lung cancer, 40,460 patients with breast cancer, and 26,180 with colorectal cancer. The most common malignancies for U.S. males during the same time period were prostate cancer (218,890), lung cancer (114,760) and colorectal cancer (79,130). Lung cancer was responsible for the highest number of deaths at 89,510, followed by prostate cancer at 27,050 and colorectal cancer at 26,000. [1].

The cost of cancer in the U.S. is staggering. For 2006, the National Institutes of Health estimated the overall cost of cancer at USD$206.3 billion. Direct medical costs accounted for USD$78.2 billion, indirect mortality costs (cost of loss productivity due to illness) USD$17.9 billion, and indirect mortality costs (cost of lost productivity due to premature death) USD$110.2 billion [1].

The treatment of cancer has revolved around three specific treatment modalities – surgery [2], radiation [3,4] and chemotherapy [5]. Depending upon the malignancy, stage at diagnosis and ultimate treatment goal (cure versus palliation) one, two or all three of these treatment modalities may be utilised.

This paper will review the innovations in radiation therapy and chemotherapy.

## RADIATION THERAPY BACKGROUND

Radiation therapy remains one of the most potent therapies in the fight against a variety of cancers. In the last decade, tremendous advances have heralded technological innovations that make treatments more precise, convenient and with less toxicity. The integration of advanced imaging such as magnetic resonance (MRI), positron emission and computed tomography (PET-CT) along with other functional imaging modalities has augmented the individualisation of each patient’s radiation therapy plan. In doing so, each patient’s unique plan can be optimised to meet the individual goals of treatment.

In previous decades, radiation teletherapy was delivered via rudimentary techniques using radioactive sources (e.g. Cobalt-60) mounted in a gantry head. This form of radiation therapy is still widely used in many parts of the developing world today where access to reliable electricity is problematic. The rapid pace of development in Asia presents the opportunity for the implementation of advanced radiotherapeutic techniques. A multidisciplinary approach to cancer care will require education, effective screening and prevention, as well as monetary and staff investment [6].

### Radiation Therapy Delivery & Planning Systems

**Figure 1:**
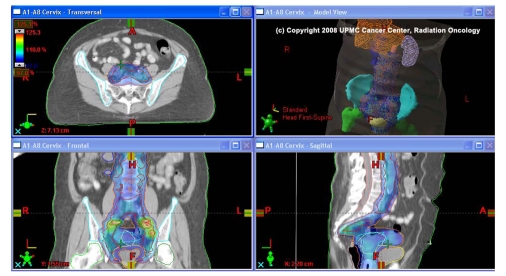
Extended-Field IMRT plan for Cervical Cancer. Note color wash radiation distribution and integrated boost treating the pathologically positive pelvic lymph nodes.

The development of the linear accelerator at Stanford University in the early 1970’s ushered in an era of highly complex plans aided by highly sophisticated computer controls [7-9]. These complex controls have facilitated the implementation of three-dimensional conformal radiation therapy (3D-CRT). The goal of 3D-CRT is to deliver a highly complex combination of radiation beam angles and field shapes using CT-based dataset to define the target volumes for treatment and avoidance of nearby critical structures. Although not formally and rigorously tested in any randomised trials (versus 2-dimensional radiation therapy), this technique has resulted in a significantly improved target definition [10,11] and reduction in treatment-related complications [12-16]. The introduction of the multi-leaf collimator (MLC), a computer-controlled beam shaping device, has increased the efficiency of conformal therapy and offered several advantages over cerrobend block designs. Dynamic shaping of the radiation beam using small, mobile tungsten leaves has enabled modulation of the radiation beam. Beam modulation can be achieved by slowing the movement of the MLC in areas where larger doses are to be delivered compared to those areas that require protection (e.g. spinal cord). The result of these dose distributions in serial adjacent axial slices is the desired conformal radiation plan. The composite result of this technique is the hallmark of intensity-modulated radiation therapy (IMRT). An example of IMRT used to treat a cervical cancer with para-aortic disease is depicted in Figure 1.

Clinical outcomes of IMRT have consistently demonstrated improvement in clinical endpoints in patients with lung cancer, intracranial tumours, prostate, gynaecologic and head and neck cancers [17-31]. Although the outcomes are quite compelling, clinical implementation of IMRT remains a major rate limiting step in a variety of practice settings. Although most modern linear accelerators are equipped to deliver IMRT, very few have delivered these treatments. The main barrier to the implementation of these advanced programmes is still the lack of clinical training and professional development of the clinical staff, i.e. radiation oncologist, physicist and/or dosimetrist. The labour intensive nature of IMRT planning further compounds the implementation of these programmes in many small, medium and large centres. Furthermore, careful training and implementation of quality assurance processes are critical and should be reassessed on a routine basis.

### Imaging and Image-guided Radiation Therapy

The introduction of computed tomography in radiation therapy in the early 1980’s was used in the era of 3D image-guided radiation therapy. With advances in computing power, complex algorithms that better model the radiation beam’s interaction with tissues have further refined our ability to predict the radiation distribution and predict the likely toxicities of treatment. Accurate definition of the target volume has become increasingly important as we seek to improve locoregional control rates while reducing the toxicities typically seen with modern cancer treatments. Multi-modality fusion of various imaging modalities such as computed tomography (CT), magnetic resonance imaging (MRI), positron emission tomography (PET), single-positron emission computed tomography (SPECT), amongst others, now provides a wealth of functional, biological and physiological data. For example, this information can now be exploited to target metabolic active or hypoxic areas of tumours. The clinical impact of these systems has been reported [25, 32, 33] and can be used to design complex individualised treatment plans.

A special application of CT involves time-resolution of a moving target such as lung cancer. Compensation of physiologic organ motion is critical in highly-conformal radiation therapy. Failure to account for target or organ motion may result in unacceptable under-dosing of the cancer or fatal irradiation of an organ or critical structure. However, after accounting for these physiological motions, individualised margins can be used to significantly reduce complications of treatment by reducing the dose delivered to uninvolved tissues while offering the opportunity to improve local control rates [13-36]. Four-dimensional computed tomography (4D-CT) now allows clinicians to better assess the tumour or organ’s trajectory in near “real-time” so that each patient’s radiation plan can be individualised to the characteristics of each situation.

Functional imaging such as magnetic resonance spectroscopy (MRS) and positron emission tomography-computed tomography (PET-CT) has opened new frontiers in the characterisation of tumours and their unique features. For example, in PET-CT imaging, the metabolic function using fluorodeoxyglucose (FDG), hypoxia using F-Misonidazole (F-Miso) or proliferation using fluoro-thymidine (FLT) can provide valuable targets for which targeted systemic agents or radiotherapeutic techniques. A summary of PET-CT tracers can be found in Table 1.

**Table 1:** PET tracers for metabolic imaging.

PET radiotracer	Function	Disease
^18^F-fluorodeoxyglucose(^18^F-FDG)	Glucose metabolism	All tumours
^11^C-methionine (^11^C-MET)	Amino acid metabolism	Brain/H&N/breast/lung/GU
^11^C-tyrosine (^11^C-TYR)	Amino acid metabolism	Brain tumours
^15^C-oxygen (^15^C-O2)	Blood flow	Brain tumours
18[F]-fluoromisonidazole	hypoxia	All tumours
^15^C-carbon monoxide (^15^C-O)	Blood volume	Brain tumours
Oxygen-15 (^15^O_2_)	Oxygen metabolism	Brain tumours
^11^C-5-hydroxy tryptophan (^11^C-5-HTP)	Serotonin levels	NE/GI
^15^O-water (H_2_^15^O)	Blood flow	Thyroid tumours
^11^C-L-dihydroxyphenylalanine (^11^C-L-DOPA)	Dopamine levels	NE/pancreatic
^18^F-fluoro-2'-deoxyuridine (^18^F-FUdR)	Nucleic acid metabolism	Brain tumours

Additionally, current linear accelerator technology has rapidly evolved to incorporate on-board imaging (OBI) which allows for the daily localisation of the radiation target volume. These systems in their current iterations use either the linear accelerator’s megavoltage beam or a separate kilovoltage (kV) source add-on the gantry of the linac. This technology generates near diagnostic quality images to improve the precision and accuracy of patient positioning and treatment verification [37-39]. The most significant feature of OBI system is its ability to use the kV source to produce volumetric CT images immediately prior to each dose of radiation treatment. These acquired images can be compared to the patient’s initial CT dataset upon which their treatment plan was created and adjustments made to the patient’s position to ensure the accurate targeting of the radiation beam. Only now, with the development of these new technologies, has the door been open to near real-time adaptive radiation therapy based on target location and shape over time. There remains significant work to be done to develop guidelines on the specific timing, frequency and the degree of adjustment that should be made based on the dynamic changes that will undoubtedly be seen when image-guided radiation therapy is more broadly adopted. Additionally, despite these new tools that may allow for more precise targeting of disease, operator limitations and multi-modality image fusion registration uncertainties are clear limitations and pose challenges to the implementation of these advanced radiotherapeutic techniques.

### Implications for Modern Therapy in Asia

The World Cancer Report, the most comprehensive global examination of the disease to date, was compiled in 2003 by the World Health Organization (WHO) IARC [40]. In that report, cancer rates are projected to increase by approximately 50% by the year 2020. In Asia, the increase is expected to outpace the worldwide average at 60% to 7.1 million cases per year. The annual incidence will climb by 45% to 163 cases per 100,000 people by 2030, from 112 cases per 100,000 population in 2005. Tobacco use, alcohol abuse and the global rise in obesity have been identified as the leading cause of the expected rise in these rates. This is further complicated by a rising life expectancy and an aging population in Asia. Previously, the cancer-specific death rate was lower in developing countries such as those in Asia, predominantly due to infectious diseases. Furthermore, the mix of cancer cases appears to be different in Asia compared to the Western world.

Lung cancer, which accounts for the largest number of cancer-related deaths, is expected to increase in incidence, further outpacing gastric cancer as the second most-common cancer with 1.2 million deaths over a 10-year period from 2005-2015 [WHO IARC report] [40]. The increasing Westernisation of the Asian diet is expected to drive a similar increase in breast, rectal and colon cancer.

To treat the expected rise in incidence of cancer, more sophisticated technologies will need to be brought to bear in the fight against the cancer. The vast majority of external beam systems in the developing world, including most of Asia, use ionising radiation from a Cobalt-60 source. Although these systems are quite reliable, especially in areas lacking reliable electrical power grid, they are unable to deliver the sophisticated radiation therapy described above. As the rapid pace of development of these countries unfolds and the growth rate of cancer incidence is realised, there will be a great need to deploy these new methods into clinical practice.

### Challenges for Implementation of Advanced Cancer Care in Asia

Tatsuzki and Levin in 2001 clearly document a number of the challenges facing countries in the Far East in caring for cancer patients [41]. This survey-based report of 17 countries in South Asia, Southeast Asia, East Asia and Australia revealed large differences in equipment and personnel. For effective use of radiotherapy, there must be a planned education programme for the general public and for referring doctors. Radiotherapy must be a part of a multidisciplinary cancer care programme. The availability of teletherapy and brachytherapy services were clearly based on the economic status of the countries examined. In many countries, patients were treated with CT or fluoroscopy-based systems. Treatment planning is also quite basic and often was performed without computers. The availability of radiation oncologists or cancer specialists further complicates the disparity of cancer care often encountered in these countries [42].

### Radiation Summary

The field of radiation oncology has undergone a remarkable transformation in technology and processes that have enhanced the therapeutic ratio since the discovery of x-rays nearly 100 years ago. As an increasing number of countries in the Far East encounter the increasing incidence of a variety of cancers, the implementation of advanced radiotherapeutic techniques will be critical in reducing treatment-related toxicities. There is a great need for professional staff training if the potential of these advances are to be fully realised.

## CHEMOTHERAPY BACKGROUND

Chemotherapy has changed a great deal over the past several years due to an improved understanding of the human genome as well as the molecular nature of cancer.

Treatment even in the early twenty-first century centered around the administration of large doses of cytotoxic chemotherapy agent(s) that affected any actively dividing cell within the body whether it be malignant or normal [43]. In some cases, this led to the eradication of the cancer but not without significant toxicity including life-threatening neutropenia and thrombocytopenia, mucositis, alopecia and neuropathy [44].

### Targeted Therapy

Targeted chemotherapy for the treatment of a malignancy was initiated with the US Food and Drug Administration (FDA) approval of trastuzumab (Herceptin®) in September of 1998. Targeted chemotherapy drugs specifically attack the malignant cell line, leaving most normal cells unharmed. These therapies can be more effective and cause fewer side effects than standard cytotoxic chemotherapeutic agents. Trastuzumab is a humanised IgG1-kappa monoclonal antibody that selectively binds with high affinity to the extracellular domain of the human epidermal growth factor receptor 2 protein or HER-2 [45]. HER-2 has been found to be over-expressed in 20-25% of all women with breast cancer. Trastuzumab is a mediator of anti-body-dependent cellular cytotoxicity (ADCC). This trastuzumab-mediated ADCC has been shown to be preferentially exerted on HER-2 over-expressing cancer cells compared to other cells, including cancer cells that do not over-express HER2. The specificity of trastuzumab for cells which over-express HER-2 dramatically decreases the toxicity of the drug to normal cells. The common side effects associated with the administration of non-specific chemotherapeutic agents such as myelosuppression, mucositis and alopecia are not associated with the administration of trastuzumab. That is not to say that the drug is devoid of all toxicity, however. Infusion reactions, including dyspnea, hypotension and anaphylaxis have been reported with the use of the drug and all monoclonal antibodies, but are rare and manageable. Cardiomyopathy as evidenced by sub-clinical and clinical congestive heart failure and a decrease in left ventricular ejection fraction (LVEF) has been noted particularly in patients who receive trastuzumab with a concurrently administered anthracycline such as doxorubicin, an agent with recognised cardiac toxicity [45]. The cardiomyopathy associated with patients receiving an anthracycline in combination with trastuzumab is a late complication of therapy. While the dose-dependent cardiac toxicity of anthracyclines is well established [46], it is unclear at this point in time whether the same can be said for the continued use of single agent trastuzumab. A review of the spectrum and reversibility of the cardiotoxicity observed in the adjuvant trastuzumab trials was recently reported [47]. Up to 4% of patients enrolled onto adjuvant trastuzumab trials experienced severe congestive heart failure during treatment with a larger proportion of patients experiencing a sustained decrease in their left ventricular ejection fraction to less than 50%. Studies are ongoing to better characterise whether or not this is a dose-dependent event.

Lapatinib [48] is an oral tyrosine kinase inhibitor (TKI) which acts at both the epidermal growth factor (ErbB1) and the HER2 (ErbB2) receptor sites. It binds reversibly to tyrosine kinase blocking phosphorylation and activation of downstream secondary messengers thereby regulating the proliferation and survival in ErbB1 and HER2 expressing tumours. It is currently FDA-approved in combination with the oral pyrimidine analogue capecitabine for the treatment of patients with HER2 over-expressing advanced or metastatic breast cancer who have received previous therapy with an anthracycline, a taxane and trastuzumab [49].

Lapatinib, being a small molecule, has been shown to penetrate the blood brain barrier and is now being evaluated as a potential treatment in patients with metastatic breast cancer with brain involvement [50].

Phase I data on the combination of trastuzumab and lapatinib has recently been published [51]. Since trastuzumab targets the extracellular domain of HER2 (ErbB2) and lapatinib acts intracellularly with specificity for both the ErbB1 and ErbB2 receptors, this combination may prove to be of value in patients with HER2 (+) breast cancer. Patients were treated with escalating doses of lapatinib, 750 to 1500 mg administered orally once daily, in combination with trastuzumab at the standard dose of a 4 mg/kg IV loading dose followed by a 2 mg/kg weekly maintenance dose. The primary endpoint of the study was to assess the safety, clinical feasibility, optimally tolerated regimen (OTR), pharmacokinetics and preliminary activity. The OTR of the combination was lapatinib 1000 mg per day with standard weekly trastuzumab. The most frequent grade 3 drug related toxicities were diarrhoea, fatigue and rash. Of the 54 patients treated, all of whom were heavily pretreated, one patient achieved a complete response and an additional seven patients had a partial response. The authors concluded that the combination of trastuzumab and lapatinib at the doses listed above was well tolerated and clinically active in this patient population.

O’Shaughnessy *et al*. conducted the first Phase III randomised open-labeled, multi-centered study evaluating lapatinib versus lapatinib + trastuzumab in heavily pre-treated women with HER2+ metastatic breast cancer. Preliminary results were reported at the 2008 American Society of Clinical Oncology (ASCO) meeting in Chicago [52]. Two hundred and ninety six women were randomised and the average prior number of chemotherapy regimens was 6. Progression-free survival (12.0 weeks vs. 8.4 weeks; p=0.029) and clinical benefit at 24 weeks (25.2% vs. 13.2%; p=0.020) both favoured the combination therapy. There was also a trend towards a higher response rate (10.3% vs. 6.9%) and overall survival (51.6 weeks vs. 39 weeks) in those patients treated with the combination of lapatinib and trastuzumab. The full manuscript of this important study will be published in the near future.

An investigational agent that shows great promise in the treatment of a number of malignancies is pertuzumab [53]. It is a recombinant humanised monoclonal antibody which binds to the extracellular domain II of the HER2 receptor and blocks its ability to dimerise with other HER receptors. Dimerisation, or the pairing with other receptor proteins, is essential for HER receptor activity and may well play a role in the growth and survival of cancer cells. This activity is distinctly different from other monoclonal antibodies such as trastuzumab or the tyrosine kinase inhibitors such as erlotinib. The Phase I study of the drug in patients with advanced cancer has shown the drug to be well tolerated, have clinical activity in a variety of tumour types and a pharmacokinetic profile which allows for a 3-week dosing interval. Additional studies are ongoing particularly in patients with metastatic breast cancer.

Other malignancies which have been treated very effectively with targeted chemotherapy include chronic myelogenous leukaemia (CML) and gastrointestinal stromal tumours (GIST), both with the oral agent imatinib mesylate.

A list of other FDA approved targeted therapy for the treatment of cancer in the US is shown in Table 2.

**Table 2:** FDA approved targeted therapy for the treatment of cancer in the US

**Extra-Cellular Receptor Blockage**			
**Drug**	**Mechanism of Action**	**Disease**	**Toxicity**
Alemtuzumab [54]	Blocks CD52	CLL/Lymphoma	Myelosuppression
Cetuximab[55]	Blocks EGFR	Colon cancer	Skin rash
Panitubumab[56]	Blocks EGFR	Colon cancer	Skin rash
Rituximab [57]	Blocks CD20	Lymphoma	Infusion reactions
Trastuzumab[58]	Blocks HER2	Breast Cancer	Cardiac
**Neutralizes Extra-Cellular Growth Factors**			
**Drug**	**Mechanism of Action**	**Disease**	**Toxicity**
Bevacizumab [59]	Binds to VEGF	Colon, Lung, Breast cancer	Skin rash
**Intra-Cellular Tyrosine Kinase Inhibitors**			
**Drug**	**Mechanism of Action**	**Disease**	**Toxicity**
Dasatinib [60]	Inhibits bcr-abl TK	CML	Fluid retention
Gefitinib [61]	Inhibits TK	Lung cancer	Skin rash
Imatinib [62]	Inhibits bcr-abl TK	CML	Fluid retention
**Intra-Cellular EGFR (HER-1) Inhibitors**			
**Drug**	**Mechanism of Action**	**Disease**	**Toxicity**
Erlotinib [63]	Inhibits HER-1	Lung cancer	Skin Rash
**Intra-Cellular Tyrosine Kinase/EGFR Inhibitors**			
**Drug**	**Mechanism of Action**	**Disease**	**Toxicity**
Lapatinib [48]	TK/EGFR Inhibitor	Breast cancer	Hand-Foot Syndrome
**Intra-Cellular m-TOR Inhibitors**			
**Drug**	**Mechanism of Action**	**Disease**	**Toxicity**
Temsirolimus [64]	m-TOR inhibitor	Renal cancer	Myelosuppression
**Intra-Cellular Multi-Kinase Inhibitors**			
**Drug**	**Mechanism of Action**	**Disease**	**Toxicity**
Sunitinib [64]	Multi-TK Inhibitor	Renal cancer	Diarrhoea
**Intra-Cellular and Surface Kinase Inhibitors**			
**Drug**	**Mechanism of Action**	**Disease**	**Toxicity**
Sorafenib [64]	Multi-TK Inhibitor	Renal Cancer	Diarrhoea
**Intra-Cellular Proteasome Inhibitors**			
**Drug**	**Mechanism of Action**	**Disease**	**Toxicity**
Bortezomib [65]	Proteasome Inhibitor	Myeloma	Neurotoxicity

### Improvements in Supportive Care

Besides the dramatic increase in the number of new, targeted therapies, the majority of which have less toxicity than the older cytotoxic agents, supportive care has also improved the tolerability of chemotherapy administration. The major advances have come in the areas of anti-emetics, growth factor support and bone health.

### Anti-Emetics

New classes of anti-emetics, such as the serotonin (5-HT3) receptor antagonists and neuro-kinin 1 (NK-1) antagonists, have played a major role in decreasing the incidence and severity of chemotherapy-induced nausea and vomiting (CINV). See Table 3.

**Table 3:** New Anti-Emetic Drugs

**Serotonin (5-HT3) Receptor Antagonists**	
**Drug**	**Route of Administration**
Dolasetron [66]	IV, PO
Granisetron [66]	IV, PO
Ondansetron [66]	IV, PO
Palonosetron [66]	IV, PO
**Neurokinin-1 Antagonists**	
**Drug**	**Route of Administration**
Aprepitant [66]	IV, PO

Combination therapy with members from each of these classes along with a steroid, usually dexamethasone, have become the standard of care for patients receiving moderately or highly emetogenic chemotherapy.

### Growth Factors

Growth factor support (see Table 4), particularly white cell factors, has allowed for an increase in dose intensity which is a primary treatment end point for patients with early stage breast cancer and lymphomas and may also play a role in other malignancies. The advent of long acting factors such as pegfilgrastim have allowed for a single dose of the growth factor per cycle versus daily subcutaneous administration for 7-10 days as was usually seen with filgrastim or sargramostim. The red cell growth factors have allowed for a decrease in the need for red blood cell transfusions in patients actively being treated for their malignancies. Recent studies have shown an increase in risk of thrombosis and death if these agents are used to increase haemoglobin levels to greater than 12 gm/dl in some groups of patients not actively being treated with chemotherapy. A long-acting formulation, darbepoietin, has allowed for dosing every 2 to 3 weeks with these agents, which is an advantage over the once-a-week schedule most commonly used with epoetin alfa.

**Table 4:** Growth Factor Support

**White Cell Growth Factors**		
**Drug**	**Route of Administration**	**Frequency**
Filgrastim [67]	SQ	Daily for 7-10 days
Sargramostim [67]	SQ	Daily for 7-10 days
Pegfilgrastim [67]	SQ	Once per cycle
**Red Cell Growth Factors**		
**Drug**	**Route of Administration**	**Frequency**
Darbepoietin [68]	SQ	Every 2-3 weeks
Epoetin alfa [68]	SQ, IV	Weekly

### Bone Health

The bisphosphonates, pamidronate and zoledronic acid, are extensively used in the oncology population to treat or prevent skeletal related events secondary to a number of malignancies including breast cancer, multiple myeloma and prostate cancer. See Table 5. They are also considered to be the drugs of choice for the treatment of hypercalcemia related to malignancy.

**Table 5:** Bisphosphonates

**Treatment/Prevention of Skeletal Related Events**		
**Drug**	**Route of Administration**	**Frequency**
Pamidronate [70]	IV	Every 3-4 weeks
Zoledronic acid [70]	IV	Every 3-4 weeks
**Treatment of Hypercalcemia Secondary to Malignancy**		
**Drug**	**Route of Administration**	**Frequency**
Pamidronate [71]	IV	Every 7 days
Zoledronic acid [71]	IV	Every 7 days

### New Findings

Data was recently presented at the 2008 American Society of Clinical Oncology (ASCO) meeting in Chicago regarding an improved outcome for premenopausal women with early breast cancer treated with endocrine therapy, tamoxifen or anastrozole with goserelin, and zolendronic acid [69]. Adjuvant bisphosphonate therapy with zoledronic acid was included in two of the four treatment arms to mitigate the bone loss associated with complete ovarian suppression and to explore the antitumour effects previously demonstrated in preclinical trials. Patients were treated with oral tamoxifen and goserelin +/- zoledronic acid which was compared to oral anastrozole and goserelin +/- zoledronic acid for 3 years. At least 900 patients were treated with either tamoxifen or anastrozole. The primary endpoint of the study was disease-free survival. Secondary endpoints included overall survival and the effects of treatment on local-regional relapse. At a median follow up of 60 months, the overall 5-year disease-free survival was 94% and the overall survival was 98.2% for all patients enrolled. Patients treated with zoledronic acid had an improved disease-free survival (36% increase) and relapse-free survival (35% increase) than those patients not treated with the drug. Both findings were statistically significant. The authors concluded that zoledronic acid significantly improves clinical outcome beyond those achieved with endocrine therapy alone. The optimal dose, schedule and duration of therapy with zoledronic acid in this patient population still need to be established.

### Multi-Gene Assays to Predict Recurrence

One of the most exciting advances in the area of cancer therapy has been the development of multi-gene assays which provide vital information on patient prognosis and potential response to systemic chemotherapy which would at least be complementary to the standard pathological and immunohistochemical techniques currently in use. An example would be the Oncotype DX® assay [72]. This test is particularly useful in patients with early stage estrogen receptor (+), lymph node (-) breast cancer. Two hundred and fifty candidate genes, potentially associated with breast cancer, were initially selected from the 25,000 genes in the human genome. Three independent studies and 447 patients were studied to identify a final panel of genes that strongly correlated with recurrence-free survival. Sixteen genes were selected based upon these clinical trials along with five reference genes to normalise the expression of the cancer-related genes. The assay was tested prospectively to predict the recurrence of disease in tamoxifen-treated, node (-) breast cancer patients by the National Surgical Adjuvant Breast and Bowel Project (NSABP) [73]. Reverse-transcriptase-polymerase-chain-reaction (RT-PRC) profiles were obtained in 675 tumour blocks from patients treated on the NSABP-14 trial. The results were used to calculate a recurrence score and to determine a risk group (low, intermediate, high) for each patient studied. Patients were considered low-risk if the recurrence score was < 18, based upon a total score of 100, and high-risk with a recurrence score of ≥ 31. Intermediate-risk was defined as those patients with a recurrence score between 18 and 30. The Kaplan-Meier estimate for the patients in the low-risk group who were disease-free of distant recurrence at ten years was 93.2%. Patients in the high-risk group who were free of recurrence at 10 years was significantly less at 69.5% (p < 0.001). Additional studies such as the prospective TAILORx Study are presently underway to validate these findings [74]. In this study, patients will be assigned to one of three treatment groups based upon their risk of distant recurrence as determined by the OncoType DX® assay. Patients with an Oncotype DX® recurrence score (ODRS) of < 11 (Group 1) will receive standard hormonal therapy alone. The drug choice will be at the discretion of the treating physician. Group 2, ODRS of 11-25 will be stratified according to tumour size, menopausal status, etc and assigned to either Arm I (experimental) – patients receiving hormonal therapy as in Group 1 – or Arm II (standard) – patients receiving standard combination chemotherapy followed by hormonal therapy as in Group 1. Group 3, ODRS > 25, will receive combination chemotherapy followed by hormonal therapy as in Group 1. The primary objectives of the study are to compare the disease-free survival of women with previously resected axillary lymph node (-) cancer with an ORDS of 11-25 treated with adjuvant chemotherapy and hormonal therapy versus adjuvant hormonal therapy alone. The study was also conducted to compare the distant recurrence-free interval and overall survival in patients with an ODRS of 11-25 treated with these regimens. Secondary endpoints include determining if adjuvant hormonal therapy alone is sufficient treatment for patients with ORDS < 11 and determining their disease-free survival, recurrence-free interval and overall survival. The results of this trial may dramatically change the way in which women with early stage ER(+), node (-) breast cancer are treated.

Another microarray available for the evaluation of patients with early stage breast cancer is the MammaPrint® assay [75]. It is a 70-gene assay which focuses on proliferation, with additional genes associated with invasion, metastasis, stromal integrity and angiogenesis. Unlike the OncoType DX® assay, this test requires either fresh frozen tumour samples or tissues collected into an RNA preservative solution. It is currently offered as a prognostic test for women under the age of 61 with either estrogen receptor positive or negative, lymph node negative breast cancer. The test reports either high- or low-risk patients based upon the results of the assay. A validation study found the low-risk group having a greater than 90 percent chance of being disease-free for a minimum of five years [76].

An abstract reported at the 2007 San Antonio Breast Cancer Symposium [77] reported overall survival data at 8 years for patients either treated or not treated with chemotherapy depending upon their MammaPrint® assay score. Patients receiving chemotherapy with a good profile (n=39) had an 8-year overall survival of 95%. This compared with an overall survival of 94% in 57 patients with a good profile that did not receive chemotherapy. Patients with a poor profile (n=142) had an overall survival of 73% at 8 years. This study validates the earlier findings proving the utility of the MammaPrint® in this patient population.

The potential importance of multi-gene assays such as the Oncotype DX® assay and MammaPrint® assay adds a new dimension in the attempt to tailor chemotherapy to the individual patient and treat only those patients who would truly benefit. Ongoing studies will validate the usefulness of this type of testing.

### Summary of Advances in Chemotherapy and Supportive Care

The recent advances in the treatment of cancer – targeted therapy, more effective anti-emetics, the utilisation of growth factors, emphasis on bone health and the advent of genomic testing – have made it much easier to treat patients and achieve improved outcomes, making most malignancies chronic diseases. This trend will continue into the foreseeable future with over 250 new compounds presently in clinical trial (see Table 6). Targeted therapy, improved supportive care drugs, and genomic testing all have the potential to improve the outcomes in patients with various malignancies. There is, however, significant added cost for these new drugs and technology which may limit their approval and utilisation in some parts of Asia.

**Table 6:** Examples of Future Oncology Drug Development

Drug	Mechanism of Action	Disease
Denosumab [78]	Monoclonal Ab to RANKL	Bone loss secondary to AIs
Romiplostim [79]	Small molecule agonist of c-mpl	ITP
Eltrombopag [79]	TPO stimulating peptide	ITP
Dabigatran [80]	Oral direct thrombin inhibitor	Tx/Prevention of Thrombosis
Tremelimumab [81]	Anti-CTLA4 antibody	Melanoma
Cenersen [82]	Blocks p53	Melanoma
STA 4783 [83]	Heat shock protein	Melanoma
AMG 655 [84]	Antibody inducing apoptosis	Pancreatic cancer
ZK 219477 [85]	Epothilone	Metastatic breast cancer
BZL 101 [86]	Inhibits glycosis	Metastatic breast cancer
Tipifarnib [87]	Farnesyltransferase inhibitor	AML
Pertuzumab [53]	HER Diamerization inhibitor	Breast Cancer

### Challenges for Implementation of Advanced Cancer Care in Asia

As with any treatment for cancer, a number of potential problems need to be addressed. They include, but are not limited to, the following:

- Access to adequate health care
  - The role of alternative medicine
- Availability of newer agents
  - Patent protection
- Expense of newer agents
- Complexity of prescribed regimen
- Toxicities of therapy and ability to treat them
  - Hospitalisation
  - Access to supportive care agents
- Knowledge synthesis and transfer
- Palliative and supportive care

### Chemotherapy Summary

Chemotherapy has changed a great deal over the past 20 years. It has gone from an attempt to eradicate all dividing cells with the hope that normal cells would repair the damage more readily and with greater success than the malignant cell clones, to a better understanding of the molecular changes which malignant cells undergo. This improved understanding has helped in the development of targeted therapy that is more effective and less toxic. Improved palliative and supportive care have also added to improved response rates, overall survival and quality of life for patients with the diagnosis of cancer. Coupled with multi-gene assays which assess a patient’s risk for relapse, these help to better identify those patients who will benefit from the administration of these therapies. New drug development, with over 250 compounds in active clinical trials, will also aid in the improved survival of patients with the diagnosis. The costs of these agents, as well as the combinations in which they are used, may well dictate the success of our fight against cancer in the very near future.
